# A Continuing Mission to Optimize the Care of Older Women with Breast Cancer

**DOI:** 10.3390/geriatrics2040037

**Published:** 2017-11-20

**Authors:** Kwok-Leung Cheung

**Affiliations:** School of Medicine, University of Nottingham, Royal Derby Hospital Centre, Derby DE22 3DT, UK; kl.cheung@nottingham.ac.uk; Tel.: +44-(0)1332-724881

**Keywords:** breast cancer, older women, tumor biology, geriatric assessment, treatment

## Abstract

The majority of cases of breast cancer occur in the older population who are often un-represented in clinical trials. Given the growing ageing population globally, it becomes urgent and important to identify an optimal approach so that older women with breast cancer are neither under- or over-treated. An inter-disciplinary research program is ongoing to investigate differing tumor biology according to age, and the potential use of a geriatric assessment tool, aiming to help select older women with primary breast cancer for a personalized and optimal treatment. Full considerations of the biology of the patient’s cancer and the geriatric domains of the patient must be taken into account when making treatment decisions.

## 1. Introduction

The incidence of breast cancer increases with age. Unfortunately, research efforts have been lacking and often this population is under-represented in clinical trials. However, early evidence suggests that there are differences in tumor biology, with less aggressive phenotypes more commonly seen in older women as opposed to their younger counterparts [1]. Furthermore, life expectancy in the older population appears to be limited and further shortened by frailty and co-morbid conditions. Our group saw a need to improve clinical service and support research in this area so that older women with breast cancer would not be under- or over-treated, but would instead receive personalized and optimal treatments [2].

Over 1700 older (≥70 years) women with early operable primary breast cancer were managed between 1973 and 2010 in a dedicated specialist facility in Nottingham [3]. An archive of tumor samples from these patients was available and a clinical database with long-term follow-up information was constructed. An inter-disciplinary research program began a decade ago with a theme to optimize the care of older women with primary breast cancer, now extending to beyond Nottingham. This short communication will highlight some key findings thus far, with a view to inspire further research and collaborations, while we continue to achieve our mission.

## 2. Materials and Methods

Two streams of this research program are now in place—the first one focusing on biological assessment and the second on geriatric assessment. Patients included are females with operable early primary breast cancer diagnosed at ≥70 years.

Briefly, the first stream investigates the relationship between tumor biology and clinical outcome. Materials are based on a consecutive series of 1700+ patients mentioned above, with clinical follow-up data and biological assessment of a large panel of biomarkers using tissue microarrays constructed from the tumor samples in the archive, in those patients who underwent surgery. Some patients received non-operative treatment because they were frail or chose not to undergo surgery. In order to demonstrate the impact of age on biology and clinical outcome, the work includes comparison with a previously characterized series of a similar size but on younger (<70 years) patients.

The second stream involves a prospective multi-center study in three hospitals with specialist breast units. Patients who have consented to participate undertake a series of assessments using a cancer-specific comprehensive geriatric assessment (CGA) tool [4], an established tool for quality of life (QOL) assessment, a semi-structured interview, at diagnosis and also at six months. Approximately 100 patients have been recruited thus far. As far as this study is concerned, demented patients are not included.

Ethical approval was obtained to conduct both studies from Nottingham Research Ethics Committee (reference numbers C1080301 and 09/H0403/12 for streams 1 and 2 respectively).

## 3. Results

### 3.1. Stream 1—Biological Assessment

The key findings from this stream include the following.

- Histological type [5]: Older patients with invasive ductal carcinomas had better survival outcomes, when compared to their younger counterparts [6]. Such difference was not observed in non-ductal carcinomas.
- Subtypes: The low estrogen receptor (ER) luminal cluster (with low expression of ER and high expression of luminal cytokeratins), distinct in older patients, was identified, adding to the five known subtypes (luminal A, luminal B, basal, normal-like and HER2 over-expressing) [5].
- ER positive tumors: Older patients have a greater preponderance of highly ER positive tumors [7]. The degree of ER-positivity showed differentiation in the effectiveness of surgery versus primary endocrine therapy. There was no difference in breast cancer specific survival (BCSS) between these treatments if patients had tumors with a histochemical (H) score ≥ 250 (out of 300) [8].
- HER2 positive tumors [9]: Tumors in older patients showed lower Ki67 and higher bcl2 expression. Only 26% of the younger patients and none of the older patients received adjuvant chemotherapy, and no patients at the time received trastuzumab. However, there was no difference in BCSS.
- Triple negative tumors [10]: Tumors in older patients showed lower Ki67 and CK 7/8 positivity and higher bcl2 and CK18 positivity. No difference in clinical outcomes existed between the two age groups, although 47% of the younger patients had adjuvant chemotherapy, while none in the older cohort received chemotherapy.

### 3.2. Stream 2—Geriatric Assessment

Stream 2 started later than stream 1. Based on the pilot study (*N* = 47) done in Nottingham, i.e., the first of the three centers, CGA determined that increasing age (≥80 years), greater (≥4) comorbidity, greater number (≥4) of daily medications, and slower (≥19 s) timed up and go (TUG) score at diagnosis were significantly related to non-operative treatment [11]. Baseline QOL scores were generally good and they remained stable at six months follow-up.

## 4. Discussion

The work from this inter-disciplinary research program has thus far demonstrated differing biology of breast cancer according to age, as well as correlation of CGA scores with treatments in older women with primary breast cancer. It is therefore important to take full consideration of both tumor biology and geriatric assessment into account when making treatment decisions. Further work is ongoing to identify a better assessment tool which can effectively achieve this goal. That includes developing a novel method of assessing tumor biology on a limited amount of tumor samples obtained from diagnostic needle core biopsies for those patients who did not undergo surgery in stream 1. The impact of this kind of research on male patients should also be explored. Given the growing ageing population globally, this would have significant impact and benefits to patients, healthcare professionals and health service commissioners.
